# Uncommon Anatomic Predisposition to Myocardial Infarction: A Case of Coronary Artery Ectasia

**DOI:** 10.7759/cureus.9035

**Published:** 2020-07-06

**Authors:** Amre Ghazzal, Laith Ali, Sohab Radwan, Gauravpal S Gill, Hector M Garcia-Garcia

**Affiliations:** 1 Internal Medicine, MedStar Washington Hospital Center, Washington, DC, USA; 2 Cardiology, MedStar Washington Hospital Center, Washington, DC, USA

**Keywords:** coronary artery ectasia, acute coronary syndrome

## Abstract

Coronary artery ectasia (CAE) is a recognized cause of acute coronary syndrome (ACS), and can be associated with life-threatening complications, including thrombus formation with consequent distal coronary artery embolization. Several studies have demonstrated a higher incidence of cardiovascular adverse events and cardiac death in patients with CAE or coronary artery aneurysms compared to those without such abnormalities. Management of symptomatic CAE is similar to coronary artery disease (CAD), where guideline-directed medical therapy is indicated due to coexistence of CAD with acquired CAE. Percutaneous coronary intervention can be attempted; however, it is challenging, as it is associated with lower procedural success, higher rates of stent thrombosis, and repeat revascularization.

## Introduction

Acute coronary syndrome (ACS) describes the range of myocardial ischemic states that include unstable angina, non-ST or ST segment elevation myocardial infarction. It is associated with substantial morbidity and mortality and places a large financial burden on the health care system. Coronary artery ectasia (CAE) is a recognized cause of ACS. It can be classified as congenital or acquired, with atherosclerosis being the most common cause of acquired CAE [1]. CAE presents commonly as stable angina; however, it can present as ACS [2]. It has also been associated with life-threatening complications, including thrombus formation with consequent distal coronary artery embolization, as well as shunt formation and rupture [3]. Managing CAE is similar to coronary artery disease (CAD); however, difficulties facing stent deployment in ectatic segments and complications associated with it pose a challenge for treating physicians.

## Case presentation

**Figure 1:**
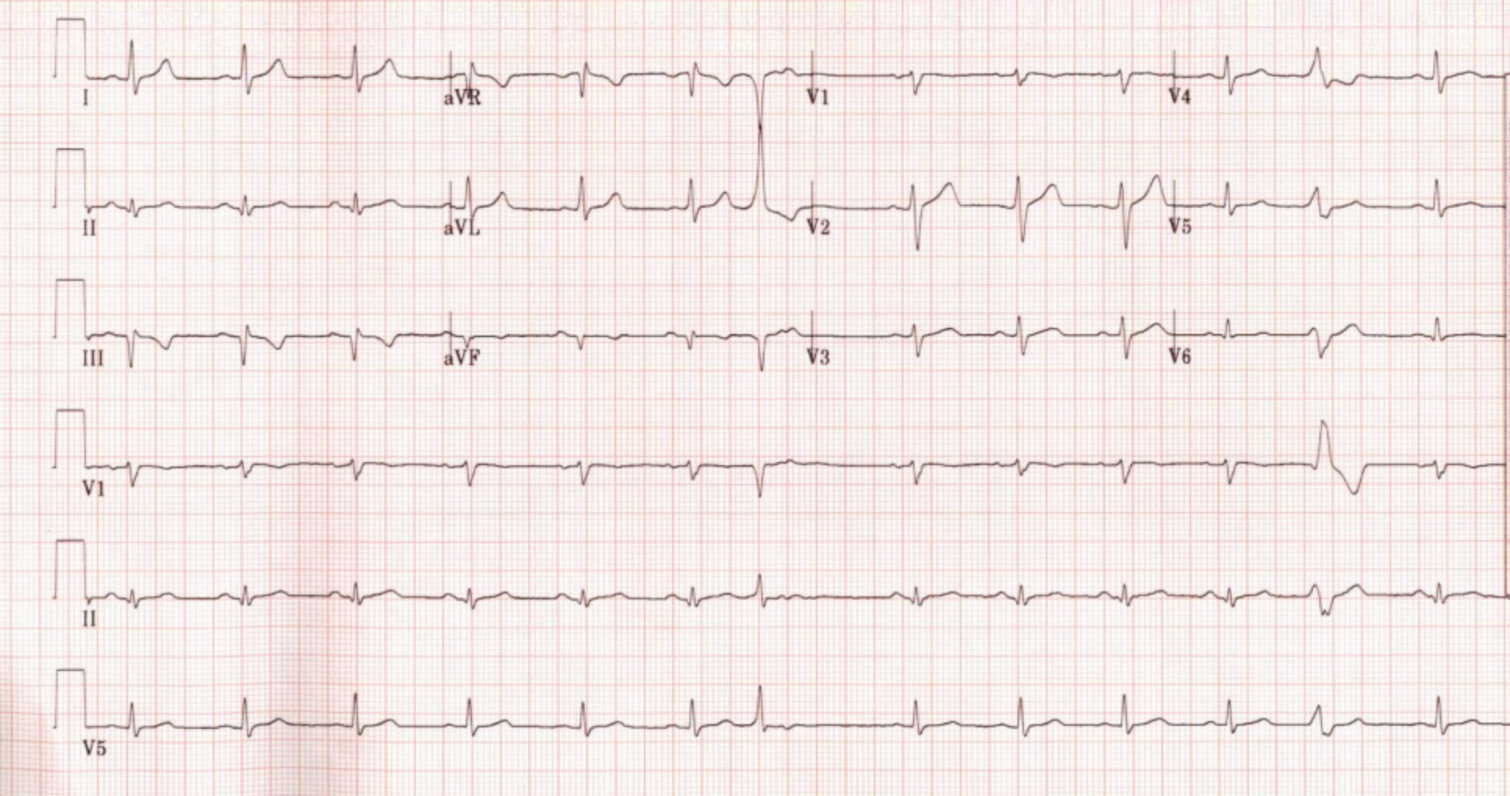
Electrocardiogram showing Q waves in leads III and aVF, T wave inversion in lead III, and premature ventricular contractions.

A 41-year-old male patient with a past medical history of hypertension and polysubstance use presents with chest pain and dyspnea on exertion of long duration. He was hemodynamically stable on presentation, and initial electrocardiogram demonstrated no significant ST-segment deviations, Q waves in leads III and aVF, T-wave inversion in lead III, and premature ventricular contractions (Figure 1); however cardiac biomarkers, namely cardiac troponin I, were elevated at 11.036 ng/mL.

**Figure 2:**
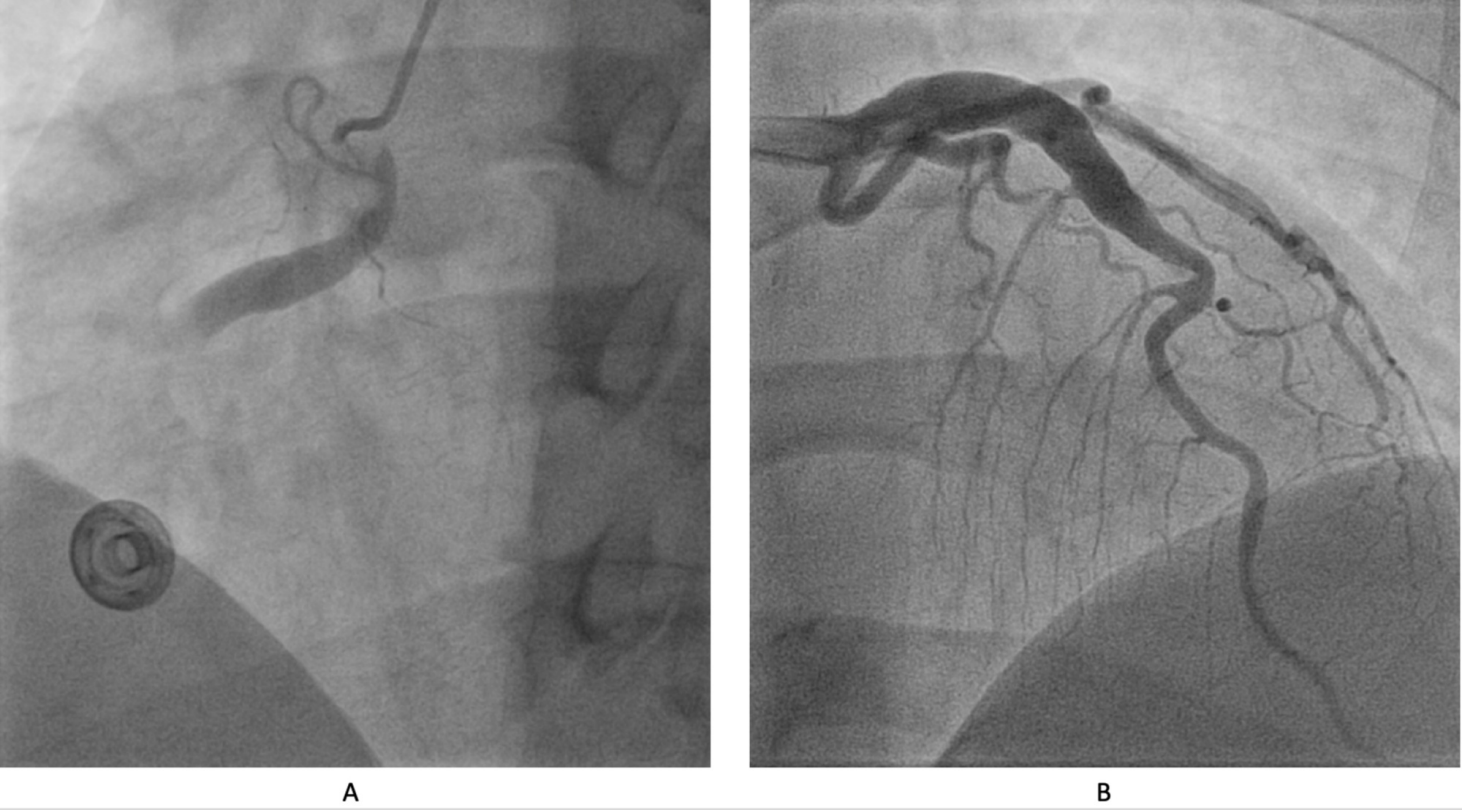
(A) Ectatic right coronary artery with obstructive intramural thrombus. (B) Ectatic left anterior descending artery.

He was medically managed with aspirin, nitroglycerin, and heparin, and admitted for further evaluation and management. Serial cardiac troponin I 6 hours later trended up to 20.10 ng/mL and due to persistent chest pain, he underwent coronary angiography which demonstrated a significantly ectatic right coronary (Figure 2A) and left anterior descending arteries (Figure 2B). There was also a thrombus in the right coronary (Figure 2A) artery posing a high risk for distal coronary embolization, stent placement was deferred, and thrombus aspiration was performed. Following the procedure, medical management with guideline-directed medical therapy was continued.

## Discussion

The terms "coronary artery ectasia" and "coronary artery aneurysm" have been used interchangeably; however, the term aneurysm describes a focal dilation of a coronary segment, while the term ectasia suggests a more diffuse aneurysmal lesion [4]. Several studies have demonstrated a higher incidence of cardiovascular adverse events and cardiac death in patients with CAE or coronary artery aneurysms as compared to those without such abnormalities [5,6].

In general, management of CAE is similar to CAD, with few differences [1]. Antiplatelet therapy is indicated for CAE due to its coexistence with CAD. This is supported by literature showing that CAE is associated with increased platelet activation due to higher expression of P-selectin, beta-thromboglobulin, and platelet factor 4 as compared to controls with angiographically normal coronaries [3]. Anticoagulation with warfarin was suggested in one study to reduce the risk of thrombus formation [7]. Moreover, statins have been found to reduce expression of metalloproteinases contributing to CAE pathophysiology, while angiotensin-converting enzyme inhibitors (ACEi) were found to reduce progression of CAE due to its association with ACEi gene polymorphism [8,9]. Nitrates, on the other hand, should be avoided as they were shown to increase exercise-induced angina [10]. If refractory symptoms persist despite medical therapy, percutaneous coronary intervention can be attempted; however, it is challenging, as it is associated with lower procedural success, higher rates of stent thrombosis, and repeat revascularization [4,11-13]. Surgical options have demonstrated good outcomes in patients who fail medical and percutaneous management [14].

## Conclusions

CAE is a recognized cause of ACS and is associated with life-threatening complications and management challenges. Currently, no guidelines are available for CAE management. Further large-scale clinical trials are needed to establish guidelines for managing CAE.
